# An Interesting Case

**Published:** 1894-01

**Authors:** 


### Editorial.

### An Interesting Case.

Have just removed from the mouth of Miss B. M—, who is eleven years of age, the first superior temporary cuspid on the left side. The incisors, bicuspids, and first and second molars, all of the permanent set erupted—this is the only temporary remaining. The permanent cuspid was erupted about one-half of the crown through the gum, about one-fourth of an inch posterior to the lateral incisor of that side, somewhat turned on its axis, it had grown and erupted in such a way as not to come in or near the root of the temporary cuspid, as a result of which the root is intact, not a particle of absorption or wasting of the root having been made; showing very clearly that the coming of the permanent tooth exercises an influence in the removal of the roots of the temporary. There was no disease of the gums near about this tooth nor any of the teeth in the mouth, everything seemed to be in a state of perfect health so far as the gums and sockets of the teeth were concerned. I have seen many such cases, but have never yet seen an instance in which the roots of the temporary teeth were absorbed when the corresponding permanent tooth was so far out of place as to have no influence upon the temporary. If in any case the roots of the temporary teeth have been found absorbed under similar conditions, or in the absence of the permanent teeth, we would be thankful to be advised of the fact.

